# Using RNA-Seq to Profile Soybean Seed Development from Fertilization to Maturity

**DOI:** 10.1371/journal.pone.0059270

**Published:** 2013-03-15

**Authors:** Sarah I. Jones, Lila O. Vodkin

**Affiliations:** Department of Crop Sciences, University of Illinois, Urbana, Illinois, United States of America; UCLA-DOE Institute for Genomics and Proteomics, United States of America

## Abstract

To understand gene expression networks leading to functional properties and compositional traits of the soybean seed, we have undertaken a detailed examination of soybean seed development from a few days post-fertilization to the mature seed using Illumina high-throughput transcriptome sequencing (RNA-Seq). RNA was sequenced from seven different stages of seed development, yielding between 12 million and 78 million sequenced transcripts. These have been aligned to the 79,000 gene models predicted from the soybean genome recently sequenced by the Department of Energy Joint Genome Institute. Over one hundred gene models were identified with high expression exclusively in young seed stages, starting at just four days after fertilization. These were annotated as being related to many basic components and processes such as histones and proline-rich proteins. Genes encoding storage proteins such as glycinin and beta-conglycinin had their highest expression levels at the stages of largest fresh weight, confirming previous knowledge that these storage products are being rapidly accumulated before the seed begins the desiccation process. Other gene models showed high expression in the dry, mature seeds, perhaps indicating the preparation of pathways needed later, in the early stages of imbibition. Many highly-expressed gene models at the dry seed stage are, as expected, annotated as hydrophilic proteins associated with low water conditions, such as late embryogenesis abundant (LEA) proteins and dehydrins, which help preserve the cellular structures and nutrients within the seed during desiccation. More significantly, the power of RNA-Seq to detect genes expressed at low levels revealed hundreds of transcription factors with notable expression in at least one stage of seed development. Results from a second biological replicate demonstrate high reproducibility of these data revealing a comprehensive view of the transciptome of seed development in the cultivar Williams, the reference cultivar for the first soybean genome sequence.

## Introduction

Soybean is one of the most important oilseed crops in the world, used as food for both humans and animals as well as for a variety of industrial oil applications. The recent sequencing of the soybean genome [1] coupled with improvements in next-generation RNA sequencing technology allow a powerful way to study expression changes in thousands of genes throughout multiple stages of soybean seed development which influence such agronomically-important traits as oil and protein production, disease resistance, and stress tolerance. A recent increase in large scale soybean seed development studies using technologies such as microarrays [2] and cDNA sequencing [3] emphasizes the importance of understanding the genetics underlying this crop's seed development pathway.

Carlson and Lersten [4] have reviewed the events in early soybean seed development, which may vary in exact timing depending on cultivar and environmental conditions. Soybeans are largely self-fertilizing and the first day the flower is fully open is usually considered the first day of fertilization. The stage when the soybean plant begins flowering is also known as R1 [5]. Around 3–5 DAF (days after flowering) the embryo has a spherical shape (known as the globe or globular stage) with an acellular endosperm and a rudimentary vascular system developing through the outer integument (which will become the seed coat). The cotyledons initiate during the heart stage at about 8–10 DAF, by which point the endosperm is almost entirely cellular; the inner and outer integuments have thickened to ten to fifteen cell layers each.

By 12–14 DAF, approximately half of the available space in the seed is taken up by the endosperm, the cotyledons have begun to elongate, and the vascular system running through the outer integument becomes much more extensive. Around 17–19 DAF, the endosperm begins to degenerate and its cellular contents are assimilated by the cotyledons, where organelles as well as protein and lipid globules have begun to form. By 22–24 DAF, the cotyledons and primary leaves are well on their way towards their full size in the seed, and the primordium of the first trifoliate leaf begins to develop. The stages from 12 DAF to 24 DAF tend to occur between R3 and R4 in this experiment, with seeds found in pods between 5 mm and 15 mm long [5].

Later stages of soybean seed development have been well-described by [5]. Between the stages of R4 and R7 the seeds grow rapidly, accumulating nutrients and storage proteins. During R6, the vegetative parts of the parent plant start to turn yellow and leaf senescence begins. The accumulation of nutrients slows down in the seeds and by R7, virtually all the seed’s dry weight has been acquired. In the cultivar Williams used here, the total fresh weight of the seed peaks around 400–500 mg. After this point the seed starts to desiccate and turn yellow, and the total fresh weight of the seed decreases as water is lost. At this point the seed contains a large embryo with cotyledons, primary leaves, and a primordium for the first trifoliate leaf, all surrounded by a three-layer dry seed coat [4]. By stage R8, almost all of the pods and seeds have turned brown and dry and the embryos are quiescent. Later, when the seed imbibes water and begins germination, metabolic activity can restart immediately using enzymes, initiation and elongation factors, and other compounds that were produced during development and stored [6].

Previously, we examined gene expression changes during stages from mid-maturation to full maturation, the period of the most intensive seed fill, using microarrays [7]. Here, we use the power of high-throughput RNA sequencing technology, including very young stages starting at just a few days after flowering, as well as later mature seed stages, to elucidate expression patterns across a broad period of seed development revealing the entire transcriptome.

## Results

### Stages and Replicates Used

Seven stages of soybean seeds were studied, encompassing major milestones in development such as tissue differentiation, nutrient accumulation, storage protein synthesis, and desiccation as shown in Table 1. Younger stages (4 DAF, 12–14 DAF, 22–24 DAF, 5–6 mg) were examined as whole seeds (combined seed coat and cotyledon) in order to preserve the liquid endosperm without damage. Cotyledons were examined in two older stages (100–200 mg and 400–500 mg) in order to study the expression of important storage protein genes during the period of seed fill. Dry seeds were also used whole, with combined cotyledon and seed coat.

**Table 1:** Stages of soybean seed development and RNA-Seq data.

Stage	Age/Weight	Tissue	Developmental Events	Bio Rep 1 Reads	Bio Rep 2 Reads
1	4 DAF	Whole seed	Spherical embryo; acellular endosperm	11.5 M	51.1 M
2	12–14 DAF	Whole seed	Cotyledons elongate; endosperm forms cell walls	24.3 M	183.5 M
3	22–24 DAF	Whole seed	Cotyledons have organelles; first trifoliate leaf initiates	77.6 M	181.9 M
4	5–6 mg	Whole seed	Endosperm depleted; rapid accumulation of water, nutrients	30.5 M	149.9 M
5	100–200 mg	Cotyledon	Nutrient accumulation continues; storage proteins synthesized	18.1 M	129.7 M
6	400–500 mg	Cotyledon	Peak of fresh weight; embryo prepares for desiccation	34.1 M	122.0 M
7	100–200 mg	Whole seed, dry	Quiescent embryo; low moisture content	30.2 M	64.0 M

Shown are the seven stages of soybean seed development used for RNA-Seq. Days after flowering (DAF) or fresh weight in milligrams (mg) is shown along with tissue and the number of processed reads obtained from two biological replicates of RNA high-throughput sequencing (in millions, M). Key events in development are noted.

High-throughput next generation transcriptome sequencing using Illumina technology (RNA-Seq) was performed on seven stages of soybean seeds, with two biological replicates per stage, resulting in millions of reads per sample; see Table 1 for totals. These reads were aligned to 78,773 soybean gene models determined by the Soybean Genome Project [1] using the program Bowtie [8]. Thousands of genes were found to be significantly differently expressed between various stages (Table 2). Data is given in RPKMs (reads per kilobase of gene model per million mapped reads; [9]). Data from the two biological replicates are highly similar, with between 99.53% and 99.99% of gene models showing no significant difference in expression between the two biological replicates of a given stage (Table 3). The RPKM filtering and data clustering were performed using values from Biological Replicate 1; the values from Biological Replicate 2 of the specific gene models in each cluster were graphed to show the overall similarity of the expression trends between the two replicates.

**Table 2:** Significantly different gene models between two stages of soybean seed development.

Stages Compared	Number of Gene Models	Percentage of Gene Models
4 DAF vs. 5–6 mg whole seed	3435	4.4
100–200 mg cotyledon vs. dry whole seed	10594	13.5

Number and percentage (of 78,773) of gene models significantly different in expression between two stages of soybean seed development (adjusted p-value ≤0.05). Data from two biological replicates were used for each stage compared.

**Table 3:** Gene models with no significant difference between biological replicates.

Stage	Number of Gene Models	Percentage of Gene Models
4 DAF	78560	99.73
12–14 DAF	78732	99.95
22–24 DAF	78660	99.86
5–6 mg whole seed	78402	99.53
100–200 mg cotyledon	78762	99.99
400–500 mg cotyledon	78760	99.98
Dry whole seed	78717	99.93

Number and percentage (of 78,773) of gene models with no significant difference in expression between two biological replicates (adjusted p-value >0.05), for each stage of soybean seed development.

### Gene Models Expressed Highly in Young Seeds Only

The 78,773 soybean gene models were filtered to retain gene models with RPKM≥500 in at least one of the four young stages (4 DAF, 12–14 DAF, 22–24 DAF, 5–6 mg) and also RPKM <500 in all three older stages (100–200 mg cotyledon, 400–500 mg cotyledon, dry seed). This resulted in a list of 111 genes with a peak in expression in at least one of the young seed stages, and lower expression in all older seed stages. The minimum RPKM of 500 was chosen to highlight the most highly-expressed genes at these early seed development stages. These gene models were then grouped into 6 clusters with Multi-Experiment Viewer (MeV; [10]). All six clusters are shown in Figure S1 and the gene models in each cluster are listed in Table S1.

**Figure 1:**
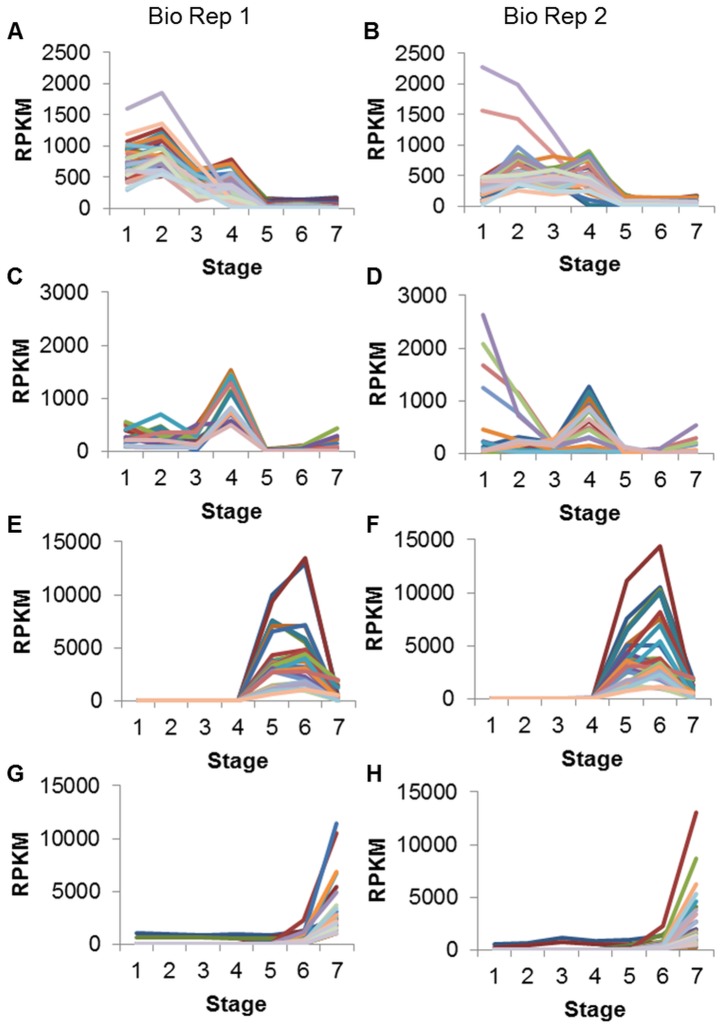
Clusters of gene models prominent at different developmental stages in soybean seeds. Clusters were created from Biological Replicate 1 data (A, C, E, G) and the same gene models were also graphed with Biological Replicate 2 data (B, D, F, H). A and C have gene models with RPKM≥500 in at least one of four young seed stages, and RPKM <500 in all three older stages. E and G have gene models with RPKM≥1000 in at least one of three older seed stages. Stages are numbered in order on the x axis: 4 DAF whole seed, 12–14 DAF whole seed, 22–24 DAF whole seed, 5–6 mg whole seed, 100–200 mg cotyledon, 400–500 mg cotyledon, dry whole seed.

The cluster in Figure 1A contains 41 gene models. In general these genes peak in RPKM at 12–14 DAF whole seed, with RPKMs ranging from about 500 to over 1800. There is also a secondary peak at 5–6 mg whole seed, with most RPKMs ranging from about 200 to 800. Throughout the three oldest stages of development RPKMs are <200. Of the 41 genes in Figure 1A, 26 models (63.4%) are annotated as histones (Table 4). Other gene model annotations found in this cluster include chlorophyll A-B binding protein and protease inhibitor/seed storage/LTP family. RPKM data for these 41 genes from Biological Replicate 2 (Figure 1B) also show higher expression in the younger seed stages than in the older.

**Table 4:** Annotations for gene models shown in Figure 1.

Annotation	Number of Gene Models	Percentage of Gene Models
*Figure 1A* * (high in young seeds)*	*41 total*	
PF00125 Core histone H2A/H2B/H3/H4	26	63.4
PF00504 Chlorophyll A-B binding protein	3	7.3
PF00234 Protease inhibitor/seed storage/LTP family	3	7.3
PF02704 Gibberellin regulated protein	2	4.9
PF08069 Ribosomal S13/S15 N-terminal domain	2	4.9
Other	5	12.2
*Figure 1C* * (high in young seeds)*	*20 total*	
AAA66288.1 proline-rich protein	6	30.0
Unknown function	5	25.0
ACA04850.1 senescence-associated protein [Picea abies]	3	15.0
CAB45653.1 putative tonoplast intrinsic protein [P. sativum]	2	10.0
Other	4	20.0
*Figure 1E* * (high in mid-maturation seeds)*	*30 total*	
P04405.2 GLYG2_SOYBN Glycinin; P25974.1 GLCB_SOYBN Beta-conglycinin	10	33.3
CAA49340.1 ADR6 [Glycine max]	5	16.7
PF01277 Oleosin	5	16.7
PF00305 Lipoxygenase	3	10.0
Other	7	23.3
*Figure 1G* * (high in dry seeds)*	*40 total*	
PF03760 Late embryogenesis abundant (LEA) group 1	14	35.0
PF04927 Seed maturation protein	12	30.0
PF00257 Dehydrin	4	10.0
S71562 drought-induced protein SDi-6	2	5.0
PF00009 Elongation factor Tu GTP binding domain	2	5.0
ACU18354.1 unknown [Glycine max]	2	5.0
Other	4	10.0

The gene models in clusters shown in Figure 1, divided into representative PFAM and NCBI non-redundant database annotation groups along with the number and percentage (of the cluster’s total) of gene models in each group. The group “Other” represents a miscellaneous category of remaining annotations. Annotations may be shortened for clarity.

The cluster in Figure 1C contains 20 gene models whose common feature is a peak in RPKM at the 5–6mg whole seed stage. RPKMs at this stage range from about 500 to 1500. RPKMs are generally lower at the other stages of development; some gene models have a smaller peak at 12–14 DAF and/or increase slightly in RPKM at the dry seed stage, though the highest RPKM is still <500. Of the 20 gene models in Figure 1C, 6 models (30%) are annotated as proline-rich proteins (Table 4). Another 25% of genes in this cluster have unknown functions. Other gene model annotations with this expression pattern include senescence-associated proteins and tonoplast intrinsic proteins. These genes show a similar pattern of expression in Biological Replicate 2 (Figure 1D), with some displaying even higher RPKMs at the 4 DAF stage.

These 20 gene models were further examined for their RPKM values in two additional samples, 5–6 mg seed coats and 5–6 mg cotyledons (Table 5). Whereas the original 5–6 mg sample represented the combined seed coat and cotyledon, the two new samples separate those two major tissues to more precisely determine the location of gene expression. Nine of the gene models (including a 7-member splice variant family of extensin/proline-rich proteins) have RPKMs at least 100-fold higher in the 5–6 mg seed coat sample than in the cotyledon sample, with average RPKMs of about 580 and 3, respectively.

**Table 5:** Expression patterns of gene models shown in Figure 1C.

Expression Pattern	Number of Gene Models	5–6 mg Whole Seed RPKM Ave	5–6 mg Seed Coat RPKM Ave	5–6 mg Cotyledon RPKM Ave	Predominant Annotation
Much higher in seed coat	9	906	586	2.5	Extensin/proline-rich proteins
Higher in seed coat	4	870	407	50	Unknown
High in both seed coat and cotyledon	5	1128	1714	1020	Senescence-associated proteins
Higher in cotyledon	2	641	65	638	Membrane intrinsic proteins

The 20 gene models in Figure 1C (genes with high RPKM at 5–6 mg whole seed) divided into groups based on their expression patterns in the 5–6 mg separated seed coat and cotyledon samples. Data from 5–6 mg combined whole seed sample also shown. RPKMs have been averaged for each group of gene models. The predominant annotation of each expression pattern group is also shown.

Four genes, mostly with unknown functions, have higher RPKMs in the seed coat but the cotyledon RPKMs are not insignificant. The RPKMs in the seed coat average about 400, while those in the cotyledon average about 50, between 6- and 15-fold higher expression in the seed coats.

Five genes, including those annotated as senescence-associated, have high RPKMs in both the seed coat and the cotyledon at the 5–6 mg stage, with the average RPKM in both tissues being over 1000. The RPKMs in each tissue tend to be at similar levels, with most less than 2-fold higher in the seed coat. Finally, a two-member splice variant family, annotated as intrinsic proteins, displays about 10-fold higher expression in the cotyledon than in the seed coat, with average RPKMs of about 640 and 65, respectively.

### Gene Models Expressed Highly In Older Seed Stages

In another analysis, RPKMs from the 78,773 soybean gene models were filtered to include only those with RPKM≥1000 in at least one of the three older seed stages (100–200 mg cotyledon, 400–500 mg cotyledon, dry seed). A higher minimum RPKM was chosen due to the generally larger RPKMs associated with older seed stages, compared to the younger stages. This filtering resulted in a list of 162 gene models with high expression in at least one older seed stage. The genes were then grouped into 7 clusters with Multi-Experiment Viewer. All seven clusters are shown in Figure S2 and the gene models in each cluster are listed in Table S1.

The cluster in Figure 1E contains 30 genes with high RPKMs at 100–200 mg cotyledon and 400–500 mg cotyledon, and comparatively low RPKMs at all other stages. The peaks at the two cotyledon stages are roughly equal and the RPKMs range from about 1000 to 13,000. At dry seed, the RPKM may reach as high as 2000; however, throughout the four earliest stages of seed development, the highest RPKM is only 87. Of the 30 gene models in Figure 1E, 10 models (33.3%) are annotated as storage proteins such as glycinin and beta-conglycinin (Table 4). Other gene model annotations found in this cluster include auxin down-regulated (ADR) proteins, oleosin, and lipoxygenase. These same genes have a very similar expression pattern in Biological Replicate 2 (Figure 1F).

Another cluster, Figure 1G **,** contains 40 gene models. These genes peak in expression at the final stage studied, that of dry, whole seed, with RPKMs ranging from about 1000 to 11,000 at this stage. Expression is comparatively low and flat at all other stages. Of the 40 genes in Figure 1G, 14 (35%) are annotated as late embryogenesis abundant (LEA) proteins (Table 4). Another 30% are annotated as various seed proteins such as seed maturation proteins and small hydrophilic plant seed proteins. Other gene model annotations found in this cluster include dehydrins and drought-induced proteins. Data from Biological Replicate 2 (Figure 1H) shows a similar expression pattern for these genes.

### Gene Models Annotated as Storage Proteins

The NCBI non-redundant database annotations for the 78,773 soybean gene models were keyword-filtered to assemble lists of gene models annotated as various soybean seed and storage proteins such as glycinin and beta-conglycinin. These sublists were then filtered to retain gene models with RPKM≥5 in at least one of the seven seed development stages studied here.

**Figure 2:**
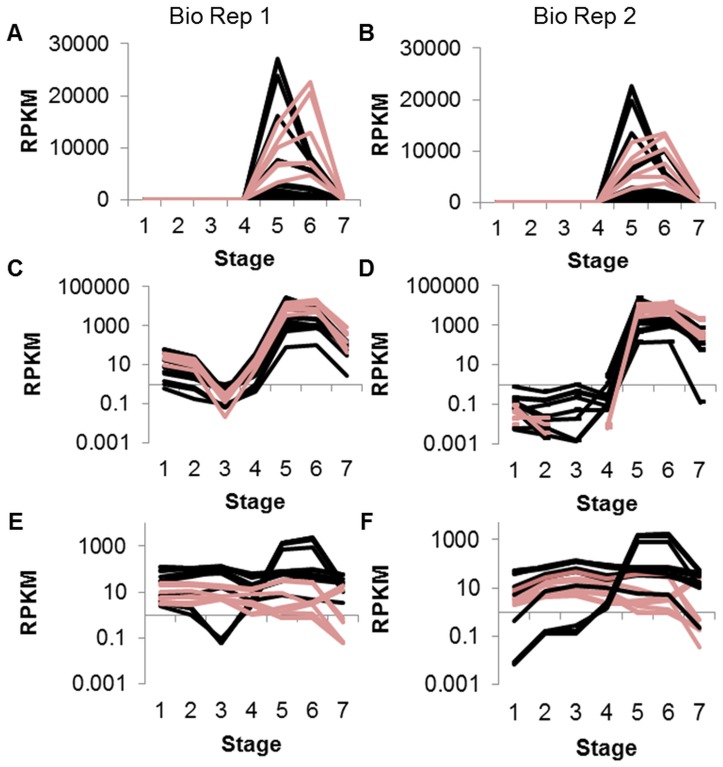
Storage protein gene models grouped by annotation. Annotations were derived from PFAM and/or the NCBI non-redundant database. Clusters were created from Biological Replicate 1 data (A, C, E) and the same gene models were also graphed with Biological Replicate 2 data (B, D, F). A shows glycinin (pink) and beta-conglycinin (black) storage proteins with RPKM≥5 in at least one of seven stages; C is the same graph using log scale. (An RPKM of 0 will not display on a log scale and may appear as a break in the line.) E shows omega-3-fatty acid desaturase (pink) and omega-6-fatty acid desaturase (black) with RPKM≥5 in at least one of seven stages, log scale. Stages are numbered in order on the x axis: 4 DAF whole seed, 12–14 DAF whole seed, 22–24 DAF whole seed, 5–6 mg whole seed, 100–200 mg cotyledon, 400–500 mg cotyledon, dry whole seed.

Glycinin and beta-conglycinin gene models with RPKM≥5 in at least one seed stage were found to have high RPKMs at both of the cotyledon stages, 100–200 mg and 400–500 mg (Figure 2A). Beta-conglycinin genes tended to peak at 100–200 mg cotyledon with most RPKMs in the range of 1000 to over 25,000. Glycinin genes, in contrast, tended to peak at the later 400–500 mg cotyledon stage, with RPKMs from about 5000 to nearly 23,000. At all other stages, RPKMs were comparatively low, with a maximum RPKM of about 60 in all four young stages (4 DAF through 5–6 mg) and a maximum RPKM of about 800 in the dry seed stage. Data for these gene models from Biological Replicate 2 showed a similar expression pattern (Figure 2B). Displaying this data on a log scale reveals variation in the expression of the genes at the younger stages of seed development (Figure 2C and, with Biological Replicate 2 data, Figure 2D).

In contrast, of the 14 gene models annotated as omega-6-fatty acid desaturase with RPKM≥5 in at least one seed stage, most were steady throughout all stages of development with RPKMs between 50 and 100 (Figure 2E). A few peaked dramatically at the two cotyledon stages, 100–200 mg and 400–500 mg, with RPKMs greater than 1000. In contrast genes annotated as omega-3-fatty acid desaturase tended to have lower RPKMs, less than 50 at most stages with a drop below 1 at the dry seed stage. A similar pattern is found in Biological Replicate 2 (Figure 2F). The specific gene models depicted in each figure are listed in Table S2.

### Gene Models Annotated as Transcription Factors

Transcription factors are key regulators of gene expression which are generally expressed at a lower level than the genes they influence. In order to explore the network of gene regulation during soybean seed development, a list was assembled of transcription factors among the 78,773 soybean gene models. The NCBI non-redundant database annotations were keyword-filtered to assemble a list of 5608 genes annotated as transcription factors, which was treated as a complete list of all transcription factor genes for the purposes of this analysis.

**Figure 3:**
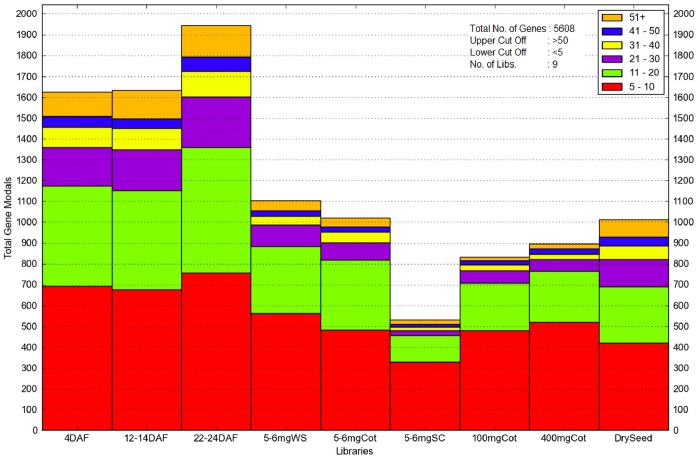
A histogram dividing 5608 transcription factor gene models into bins based on their RPKMs. Nine different libraries are shown, including 5–6 mg separated seed coat and cotyledon. Gene models must have RPKM≥5 at a stage to be displayed. Gene models with RPKM≥51 are compiled together at the top (orange). 5–6 mgWS = 5–6 mg whole seed.

The 22–24 DAF whole seed had the most transcription factors, with nearly 2000 having RPKMs≥5 (Figure 3). Roughly 40% of these genes had RPKMs in the 5–10 range, with another 30% having an RPKM of at least 21. The young whole seed samples–4 DAF, 12–14 DAF, 22–24 DAF, and 5–6 mgWS–all had more transcription factor genes (with RPKM≥5) than the three older stages, though the dry whole seed stage had only slightly fewer than 5–6 mg whole seed with about 1000 and 1100 transcription factor genes, respectively.

The fewest number of transcription factor genes (with RPKM≥5) was found in the 5–6 mg separated seed coat sample; only about 500 genes met the criteria in this tissue. Twice as many transcription factor gene models were found in the 5–6 mg separated cotyledon, only slightly fewer than in the 5–6 mg whole seed (which combines the cotyledon and seed coat tissues).

The list of 5608 transcription factor gene models was then filtered to retain genes with RPKM≥25 in at least one of the seven seed development stages studied here (not including the 5–6 mg separated seed coat and cotyledon). This minimum RPKM was chosen to highlight the transcription factor gene models with the highest expression levels throughout soybean seed development. This resulted in a list of 676 transcription factor genes with notable expression in at least one stage of seed development, about 12% of the total list of transcription factors. The 676 transcription factor gene models were then grouped into 7 clusters with Multi-Experiment Viewer. All seven clusters are shown in Figure S3 and the gene models in each cluster are listed in Table S1.

**Figure 4:**
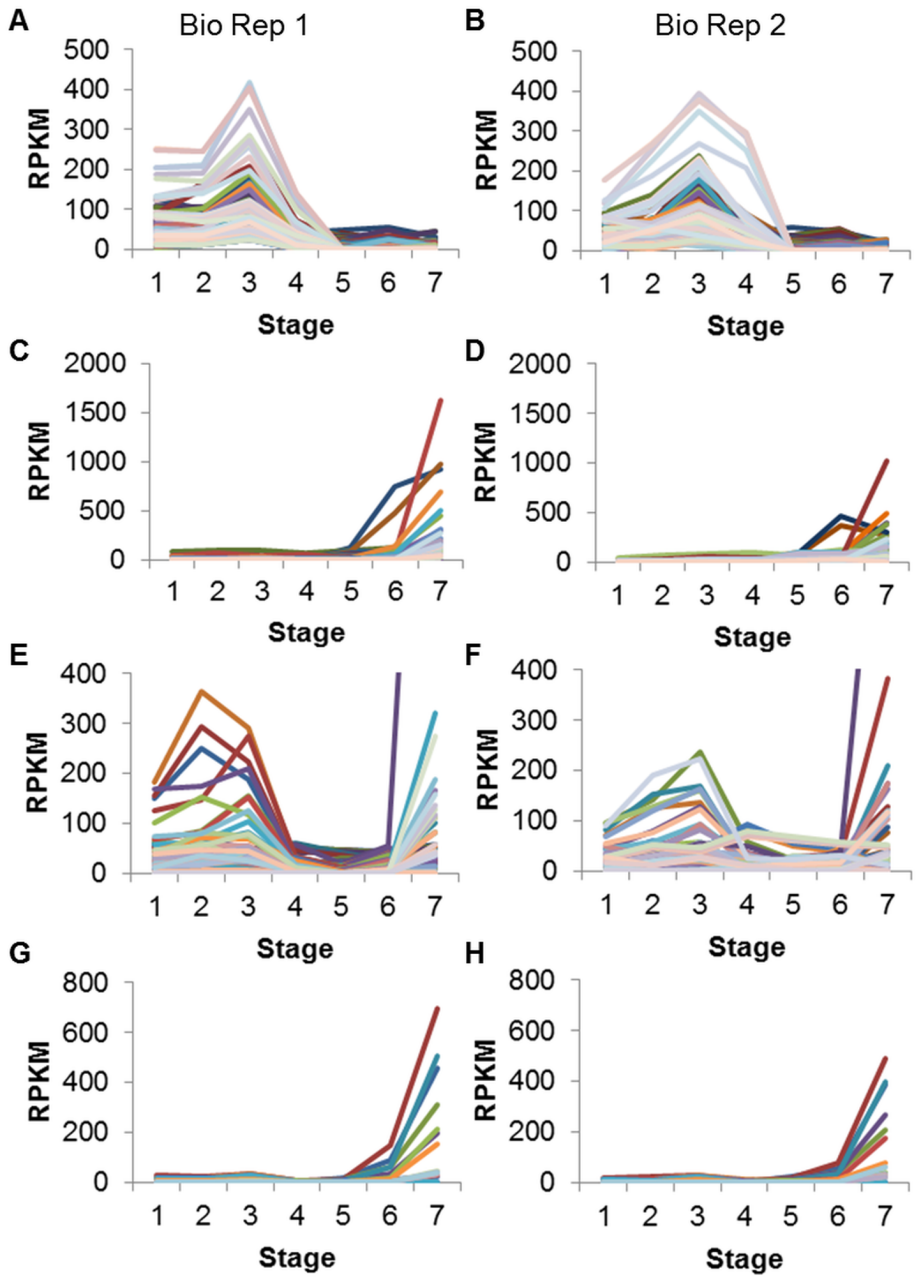
Transcription factor gene models. Clusters were created from Biological Replicate 1 data (A, C, E, G) and the same gene models were also graphed with Biological Replicate 2 data (B, D, F, H). A and C display transcription factors with RPKM≥25 in at least one of seven stages of seed development. E displays gene models annotated as AP2/ERF/EREBP (ethylene responsive) transcription factors with RPKM≥25 in at least one stage of seed development. The Y axis has been set at 400, cutting off the purple line on the right, which reaches an RPKM of over 1400 at the dry seed stage. G displays gene models annotated as No Apical Meristem (NAM/NAC) transcription factors with RPKM≥25 in at least one stage of seed development. Annotations were derived from PFAM and/or the NCBI non-redundant database. Stages are numbered in order on the x axis: 4 DAF whole seed, 12–14 DAF whole seed, 22–24 DAF whole seed, 5–6 mg whole seed, 100–200 mg cotyledon, 400–500 mg cotyledon, dry whole seed.

The cluster in Figure 4A contains 228 transcription factor genes. In general these gene models peak in RPKM at 22–24 DAF, with RPKMs ranging from about 25 to over 400. RPKMs may be notable at other young stages as well, but tend to be comparatively low at the three oldest stages. Of the 228 transcription factor genes in Figure 4A, 26 models (11.4%) are annotated as MYB-like DNA-binding domain (Table 6). Almost 10% are annotated as zinc fingers. Other gene model annotations found in this cluster include auxin response factors, AP2/ERF transcription factors, and SRF-type transcription factors. A similar pattern of expression is found for these gene models in Biological Replicate 2 (Figure 4B).

**Table 6:** Annotations for transcription factor gene models shown in Figure 4.

Annotation	Number of GeneModels	Percentage of Gene Models
*Figure 4A* * (TFs high in young seeds)*	*228 total*	
PF00249 Myb-like DNA-binding domain	26	11.4
PF00096 Zinc finger, C2H2 type	22	9.7
PF02309 AUX/IAA family; NP_851244.1 AUXIN RESPONSE FACTOR 2 [A. thaliana]	15	6.6
XP_002326298.1 AP2/ERF transcription factor	13	5.7
PF00319 SRF-type transcription factor	13	5.7
PF02362 B3 DNA binding domain	11	4.8
XP_002300324.1 global transcription factor group	11	4.8
PF02183 Homeobox associated leucine zipper	8	3.5
PF06217 GAGA binding protein-like family	7	3.1
AAY30856.1 MADS-box transcription factor [P. dulcis]	7	3.1
Other	95	41.7
*Figure 4C* * (TFs high in dry seeds)*	*108 total*	
XP_002307142.1 AP2/ERF transcription factor	21	19.4
PF02045 CCAAT-binding transcription factor (CBF-B/NF-YA)	11	10.2
PF00170 bZIP transcription factor	10	9.3
PF02365 No apical meristem (NAM) protein	10	9.3
PF00096 Zinc finger, C2H2 type	10	9.3
PF00249 Myb-like DNA-binding domain	5	4.6
CAA87076.1 heat shock transcription factor 21 [G. max]	4	3.7
Other	37	34.3

The gene models in the clusters shown in Figure 4 (transcription factors), divided into representative PFAM and NCBI non-redundant database annotation groups along with the number and percentage (of the cluster’s total) of gene models in each group. The group “Other” represents a miscellaneous category of remaining annotations. Annotations may be shortened for clarity.

The cluster in Figure 4C contains 108 transcription factor genes that generally peak in RPKM at the final, dry seed stage, with the highest RPKM being over 1600. Most of the gene models have comparatively low RPKMs at all the other stages of development, with a maximum RPKM of 125 throughout the first five stages. Of the 108 transcription factor genes in Figure 4C, 21 models (19.4%) are annotated as AP2/ERF transcription factors (Table 6). Another 10% are annotated as CCAAT-binding transcription factors. Other transcription factor annotations found in this cluster included bZIP, no apical meristem (NAM), and zinc fingers. These gene models show similar expression in Biological Replicate 2 data (Figure 4D).

The transcription factor genes can also be grouped into families according to their annotations (gene models listed in Table S2). Figure 4E shows that transcription factors annotated as AP2/ethylene responsive factors (with RPKM≥25 in at least one stage) display a variety of shapes throughout seed development. Some members of the family are most highly expressed during the early stages, such as 12–14 DAF or 22–24 DAF whole seed, while others appear to peak in expression at the final, dry seed stage. A similar variety in expression patterns is found in data from Biological Replicate 2 (Figure 4F).

The no apical meristem (NAM) transcription factors have a much more uniform expression pattern, at least those gene models with RPKM≥25 in at least one stage of seed development (Figure 4G). Most of these gene models clearly peak in expression at the dry seed stage, with the highest RPKM around 700. At the five earliest stages of seed development (4 DAF through 100–200 mg cotyledon), the highest RPKM is only about 37. This pattern of expression is repeated in Biological Replicate 2 (Figure 4H).

## Discussion

In the early stages of development, the seeds are undergoing rapid growth and differentiation as discrete structures such as the embryo, seed coat, cotyledons, endosperm, and primary leaves form. This can be seen in the annotations for genes in Figures 1A and 1C, which peak in RPKM at early stages such as 12–14 DAF and 5–6 mg (Table 4). Almost two-thirds of the gene models in Figure 1A are annotated as histones, proteins around which DNA is wound for compact storage in cells. The large number of histone genes showing high expression in the young seed stages may reflect the rapid expansion in cell number at this point in development, as new histones are produced to organize the DNA strands into nucleosomes. Histones are highly over-represented in this cluster, as a keyword search of the annotations (NCBI non-redundant database and PFAM) for 78,773 soybean gene models reveals <1% described as encoding histones.

Likewise, about 30% of the genes in Figure 1C are annotated as proline-rich proteins, which are involved in cell wall structure in plants. The high expression of these genes in early stages may be due to increased cell growth and development at this time. This annotation is also highly over-represented in this cluster, with <1% of the 78,773 soybean gene models described as encoding proline-rich proteins. Other studies have shown soybean proline-rich protein gene *PRP1* to be highly expressed in developing seed coats up to 50 mg fresh weight [11], [12].

Intriguingly, other gene models with high RPKM at these early stages are annotated as senescence-associated. For example, the genes with this annotation in Figure 1C were found to have extremely high RPKMs (>1000) in both the cotyledon/embryo and seed coat tissues of the 5–6 mg stage (Table 5). Senescence-associated plant genes and proteins have mainly been studied in the context of dying petals and leaves, tissues which release their nutrients to other parts of the plant and degrade as part of the plant’s normal life cycle [13], [14]. Our data suggest these genes have an important role in early stages of seed development as yet unexplored, especially as <1% of the total number of gene models are annotated as senescence-associated.

By the later stages of seed development, the seed is focused on accumulating nutrients and synthesizing storage proteins. The RNA-Seq data reflect this, with storage protein annotations over-represented in the gene models shown in Figure 1E, which peak at the two mid-maturation cotyledon stages. For example, common annotations (Table 4) among these genes are well-known seed storage proteins such as glycinin, beta-conglycinin, and sucrose-binding proteins, which are generally found at <1% in the genome as a whole. Similar expression patterns for these storage protein genes have been found in other studies [15]–[17]. A study by [18] of developing *Arabidopsis* seeds, starting with the very young globular stage and culminating with mature embryos and green cotyledons, also found a similar expression pattern for genes encoding oleosins and seed storage proteins.

Previous work done by our lab [7] using cDNA microarrays with the same older stages of development found that genes in the auxin down-related (ADR) and lipoxygenase families had high expression at the 400–500 mg cotyledon stage, in comparison to the 100–200 mg cotyledon stage. Lipoxygenase is involved in the storage of nitrogen and the oxidation of polyunsaturated fatty acids in seeds [19], [20] and may also be used later in reactions during early shoot growth [21]. Auxin down-regulated (ADR) genes represent an intriguing family first described by [22] as having reduced RNA concentration following auxin treatment of soybean hypocotyls. Members of this family have since been discovered to be highly expressed in soybean somatic embryos [23] and soybean cotyledons both immature [7] and post-germination [24]. Both ADR6 and lipoxygenase annotations are over-represented in this cluster, as both are found at <1% in the total list of 78,773 gene models.

A recent study [17] used Affymetrix microarrays to examine differentially expressed genes in stages of soybean seeds roughly corresponding to the middle stages of the current experiment, culminating in whole seeds at the stage of largest fresh weight for their cultivar. Genes with many of the same annotations, such as glycinin, beta-conglycinin, lipoxygenase, and oleosin, were found to peak in expression at the stage of largest fresh weight, coincident with our results. Interestingly, they also detected a number of genes annotated as seed maturation protein, late embryogenesis abundant (LEA) protein, and dehydrin with this same expression pattern; in our study such genes peaked in expression at the final, dry seed stage (Figure 1G), which [17] did not examine.

Seed storage proteins such as glycinin and omega-6-fatty acid desaturase are of great agronomic importance and have been well-studied in soybean. As shown in Figure 2A, gene models annotated as glycinin and beta-conglycinin peak in RPKM at 400–500 mg cotyledon and 100–200 mg cotyledon, respectively. Other studies [15]–[17] have found that mRNAs encoding these two major soybean storage proteins show the same pattern of low expression at early stages, followed by beta-conglycinin increasing in expression before glycinin, with a dramatic decrease for both at the mature, dry seed stage.

In contrast, most of the gene models annotated as fatty acid desaturase show steady expression throughout multiple stages of development (Figure 2E). Genes annotated as omega-6 desaturase were found to have a similar expression pattern in equivalent soybean seed stages [17], with a peak at the stage of largest fresh weight. Both omega-6 and omega-3-fatty acid desaturase are involved in the conversion of oil in the soybean seed to more highly desaturated forms throughout development.

At the final stage of development, the seed is undergoing desiccation in preparation for a quiescent period prior to imbibition and germination. Almost all of the highly-expressed gene models shown in Figure 1G are annotated as hydrophilic proteins associated with low water conditions in plants. The late embryogenesis abundant (LEA) proteins (of which dehydrins are a subfamily) are known to accumulate both in the desiccating stage of seed development as well as in vegetative tissues experiencing environmentally-induced water loss [25], [26]. These proteins may protect cellular structures in a variety of ways, such as by stabilizing cell membranes, quarantining ions that have been concentrated by water loss, or binding the remaining available water [25]. LEA genes with a similar expression pattern were found in developing *Arabidopsis* seeds, peaking in expression at the mature embryo stage [18]. The LEA and dehydrin annotations are over-represented in this cluster, as they are found in <1% of annotations in the 78,773 gene models total.

Transcription factors are crucial components of regulation systems which may initiate vital gene expression changes while displaying relatively low expression themselves. Thus when examining transcription factor gene models in this RNA-Seq data, an RPKM cut-off of just 25 was chosen. The wide variety of transcription factor families found to be highly expressed in our data reflects the complex network of regulation required during seed development, especially at the younger stages when seeds are undergoing a diverse array of morphological changes.

The transcription factor gene models shown in Figure 4A, which peak at 22–24 DAF, have many annotations (Table 6) which have also been found to be significantly expressed in developing *Arabidopsis* seeds with a similar pattern that peaks in early to middle development; such annotations include zinc finger proteins, GATA-binding factors, MYB domains, and members of the APETALA2-ethylene response factor family (AP2/ERF) [18]. MYB domain transcription factor *AtMYB123,* also known as *TRANSPARENT TESTA2*, is known to be involved in the regulation of proanthocyanidin production in developing *Arabidopsis* seed coats [27], [28]. AUX/IAA family genes and auxin response factors, similar to those also found in this cluster, interact with the hormone auxin to regulate genes involved in the proper development of young embryos and cotyledons [29]. Recently, certain MYB transcription factors as well as auxin response factors have been identified as the targets of microRNAs in multiple mature cotyledon stages of the soybean cultivar Williams (the same used in the present experiment), suggesting a mechanism by which some of these genes are down-regulated after a peak in expression at the early seed stages [30]. Small RNA profiles, including miRNAs for soybean, are available for certain stages of seed coat and cotyledon development [31], [32]. A keyword search was used to determine approximately what percentage of the 5608 transcription factor gene models fell into each family; about 14% of this larger group were annotated as Myb-like factors, suggesting this family is not over-represented in the young seed cluster with 11.4%. However, the auxin-related transcription factors comprise only about 2% of transcription factor gene models generally, but 6.6% of the cluster highly expressed at 22–24 DAF. Other families mentioned in Table 6 which are over-represented in this young seed cluster include the global transcription factor group (4.8% of the cluster), the GAGA-binding protein family (3.1% of the cluster), and MADS-box transcription factors (3.1% of the cluster), each of which comprises <1% of the transcription factors found on the larger list.

In contrast, the transcription factor gene models shown in Figure 4C peak in expression at the final, dry seed stage. Previous work from our lab [7] using cDNA and oligo microarrays with these same older stages of seed development found that an unexpectedly high percentage of transcription-related genes were expressed during the dry seed stage relative to the 100–200 mg cotyledon. The over-expressed transcription factor annotations reported included Nuclear Factor Y (NF-Y), zinc fingers, and AP2, which also appear on the current RNA-Seq list of highly expressed transcription factors at the dry seed stage (Table 6). Some of these factors could be involved in preparing transcripts in the dry seed in anticipation of germination, decreasing the time and effort needed to produce essential proteins once imbibition begins. A study by [18] of developing *Arabidopsis* seeds also using microarray data noted that *ABSCISIC ACID INSENSITIVE 5* (*ABI5*), a bZIP transcription factor, displayed a similar expression profile to Figure 4C and is known to regulate certain LEA genes, which also have this expression profile in both studies. Additionally, both NF-Y transcription factors and AP2/ERF family members have been implicated in drought responses in plants, suggesting a role they may play in desiccating seeds [33], [34]. According to the keyword classification of the list of 5608 transcription factor gene models, many of the annotations mentioned in Table 6 which peak in expression at the dry seed stage are over-represented in this cluster. For example, AP2/ethylene responsive transcription factors and the CBF/NF-Y family comprise 19.4% and 10.2% of this cluster, respectively, but <10% and <5% of the larger list. The no apical meristem (NAM/NAC) family is found at 9.3% in the cluster compared to <4% overall, and heat shock transcription factors are found at 3.7% in the cluster versus <2% overall.

It was noted that some transcription factor families, such as the AP2/ethylene responsive factors, have a variety of expression patterns throughout seed development, as different members regulate genes needed at different times (Figure 4E). This large family of transcription factors, which also includes the dehydration-responsive element binding protein (DREB) and the related to ABI3/VP1 (RAV) genes, has been linked to a similarly wide variety of biotic and abiotic stresses and hormonal signaling, as well as certain developmental processes such as floral organ identity and root morphogenesis [35]. MYB is another family in which different members are highly expressed at young stages (Figure 4A) or dry seeds (Figure 4C).

However, other families, such as the no apical meristem transcription factors, have a much more specific expression pattern, in this case being highly expressed at the final, dry seed stage (Figure 4G). NAM or NAC genes (so-called due to four early transcription factors found with the same new domain–petunia *NAM* plus *Arabidopsis ATAF1* and *2* and *CUC2)* are part of a large plant-specific family with a diverse array of functions, including biotic and abiotic stress responses, hormone signaling, leaf senescence, and meristem and primordia formation during embryogenesis [36]. A large number of the NAC genes identified in soybean, including several found in this study to have high expression in the dry, mature seeds, are known to be involved in drought stress response in other tissues such as roots and shoots [37]. This finding indicates these genes have important functions during tissue desiccation, whether brought on by environmental stress or normal developmental processes.

In this study, seven stages of soybean seeds, from just a few days after fertilization to dry, mature seeds, were subjected to next-generation sequencing, resulting in millions of sequenced transcripts per stage. When aligned to nearly 79,000 predicted soybean gene models, this wealth of data provides a broad overview of the important processes shaping the development of one of the most important oilseed crops in the world.

## Methods

### Plant Material and RNA Extraction

Immature soybean seeds (*Glycine max* cv. Williams, maturity group III) were harvested from greenhouse-grown plants. The three earliest stages were harvested at the stated Days After Flowering (DAF) and the whole seeds were removed from the early pods under an Olympus SZ61 microscope (Melville, NY) and fresh-frozen in liquid nitrogen. For the next three stages (5–6 mg, 100–200 mg, 400–500 mg), whole seeds were sorted by the stated fresh weight ranges. 5–6 mg seeds were removed from the early pods and either the whole seeds or separated seed coats and cotyledons were fresh-frozen in liquid nitrogen. For 100–200 mg and 400–500 mg seeds the separated seed coats and cotyledons were lyophilized. Dry seeds were harvested at maturity (approximately 100–200 mg) and stored whole at room temperature.

Total RNA was extracted from tissues using phenol:chloroform and a lithium chloride precipitation [24]. RNA from the three oldest stages was further purified with a Qiagen RNeasy Midi kit (Valencia, CA). Soybean is highly inbred, but in order to minimize biological variation, RNA was extracted and combined from multiple seeds and plants, using between four and one hundred different seeds depending on the stage (younger stages required more seeds be extracted at the same time to yield usable amounts of RNA). RNA from separate biological samples was used for the two biological replicates. 5–6 mg separated seed coat and cotyledon data come from only a single biological replicate.

### High-Throughput RNA Sequencing and Alignment

Total RNA was sequenced by the Keck Center (University of Illinois) using an Illumina Genome Analyzer II for the Biological Replicate 1 samples (except for 22–24 DAF), and using an Illumina HiSeq 2000 for the Biological Replicate 2 samples (and also 22–24 DAF of Bio Rep 1, 5–6 mg separated seed coat, and 5–6 mg separated cotyledon). In Biological Replicate 1, the 22–24 DAF and 100–200 mg cotyledon samples were sequenced using the single read method while the other samples were sequenced with the paired-ends method; in Biological Replicate 2, all samples used the single read method. 5–6 mg separated seed coat and cotyledon also used the single read method. For both biological replicates, as well as 5–6 mg separated seed coat and cotyledon, all samples had reads 75 bp in length after processing, except the 4 DAF and dry whole seed samples of Biological Replicate 2, which had reads 100 bp in length. The technical differences between the sample sequencing methods reflect improvements in the standard high-throughput RNA sequencing techniques over time. Sequencing data was filtered through the standard Illumina pipeline including the CASSAVA program.

The reads were aligned to 78,773 Glyma1 cDNA soybean gene models determined by the Soybean Genome Project, Department of Energy, Joint Genome Institute [1], using the alignment program Bowtie [8]. Bowtie parameters allowed up to three mismatches and up to 25 alignments per read; reads aligned more than 25 times were discarded, with none of their alignments being counted.

### Sequencing Data, Gene Model Annotations, Clustering, and P-Values

Data is given in RPKMs [9], which stands for reads per kilobase of gene model per million mapped reads. The total number of mapped reads takes into account reads that aligned up to 25 different times, including multiple matches within the same gene model.

Gene model annotations by PFAM were from the Soybean Genome Project in the Phytozome database version 4.0 [38]. Annotations of gene models using BLASTX (e-value 1e-4 or better) against NCBI’s non-redundant database version 8.0.1 have been described [39]. The top 5 hits, as well as five others from species of interest (such as *Glycine max*), were compiled for each gene model. Gene models were grouped into families by manual inspection using these annotations.

Gene models were clustered using Multi-Experiment Viewer (MeV) [10] using data from Biological Replicate 1. The k-means clustering technique was used with the Pearson correlation distance metric and 2500 maximum iterations. P-values (adjusted with the Benjamini-Hochberg procedure) both between biological replicates and between stages were generated using DESeq [40] in Bioconductor [41].

Raw and processed sequencing data are available in the Gene Expression Omnibus database at http://www.ncbi.nlm.nih.gov/geo/as series accession number GSE42871.

## Supporting Information

Figure S1 **Clusters of gene models with high expression in young seed stages.** Clusters produced using Biological Replicate 1 data (left column) depict gene models with RPKM≥500 in at least one of four young seed stages, and RPKM <500 in all three older stages. The right column shows the same gene models with Biological Replicate 2 data. Cluster numbers (determined by MeV) are shown at the far right. A and B are repeated from Figure 1C and 1D; K and L are repeated from Figure 1A and 1B. Note the different scales used by E and F. The Y axis of J has been set at 1500, cutting off the blue line, which reaches an RPKM of over 2700 at 5–6 mg whole seed. Stages are numbered in order on the x axis: 4 DAF whole seed, 12–14 DAF whole seed, 22–24 DAF whole seed, 5–6 mg whole seed, 100–200 mg cotyledon, 400–500 mg cotyledon, dry whole seed.
(TIF)

Figure S2 **Clusters of gene models with high expression in older seed stages.** Clusters produced using Biological Replicate 1 data (left column) depict gene models with RPKM≥1000 in at least one of three older seed stages. The right column shows the same gene models with Biological Replicate 2 data. Cluster numbers (determined by MeV) are shown at the far right. C and D are repeated from Figure 1G and 1H; M and N are repeated from Figure 1E and 1F. Stages are numbered in order on the x axis: 4 DAF whole seed, 12–14 DAF whole seed, 22–24 DAF whole seed, 5–6 mg whole seed, 100–200 mg cotyledon, 400–500 mg cotyledon, dry whole seed.
(TIF)

Figure S3 **All clusters of transcription factor gene models.** Clusters produced using Biological Replicate 1 data (left column) depict gene models annotated as transcription factors with RPKM≥25 in at least one of seven stages of seed development. The right column shows the same gene models with Biological Replicate 2 data. Cluster numbers (determined by MeV) are shown at the far right. Annotations were derived from PFAM and/or the NCBI non-redundant database. Stages are numbered in order on the x axis: 4 DAF whole seed, 12–14 DAF whole seed, 22–24 DAF whole seed, 5–6 mg whole seed, 100–200 mg cotyledon, 400–500 mg cotyledon, dry whole seed.
(TIF)

Table S1 **Gene models depicted in three sets of clusters.**
(XLSX)

Table S2 **Selected gene models annotated as storage proteins and transcription factors.**
(XLSX)
