# Supplementary figures and images for: Using RNA-Seq to Profile Soybean Seed Development from Fertilization to Maturity
Source: PLoS One. 2013 Mar 15;8(3):e59270. doi: 10.1371/journal.pone.0059270 (PMC3598657; doi:10.1371/journal.pone.0059270)

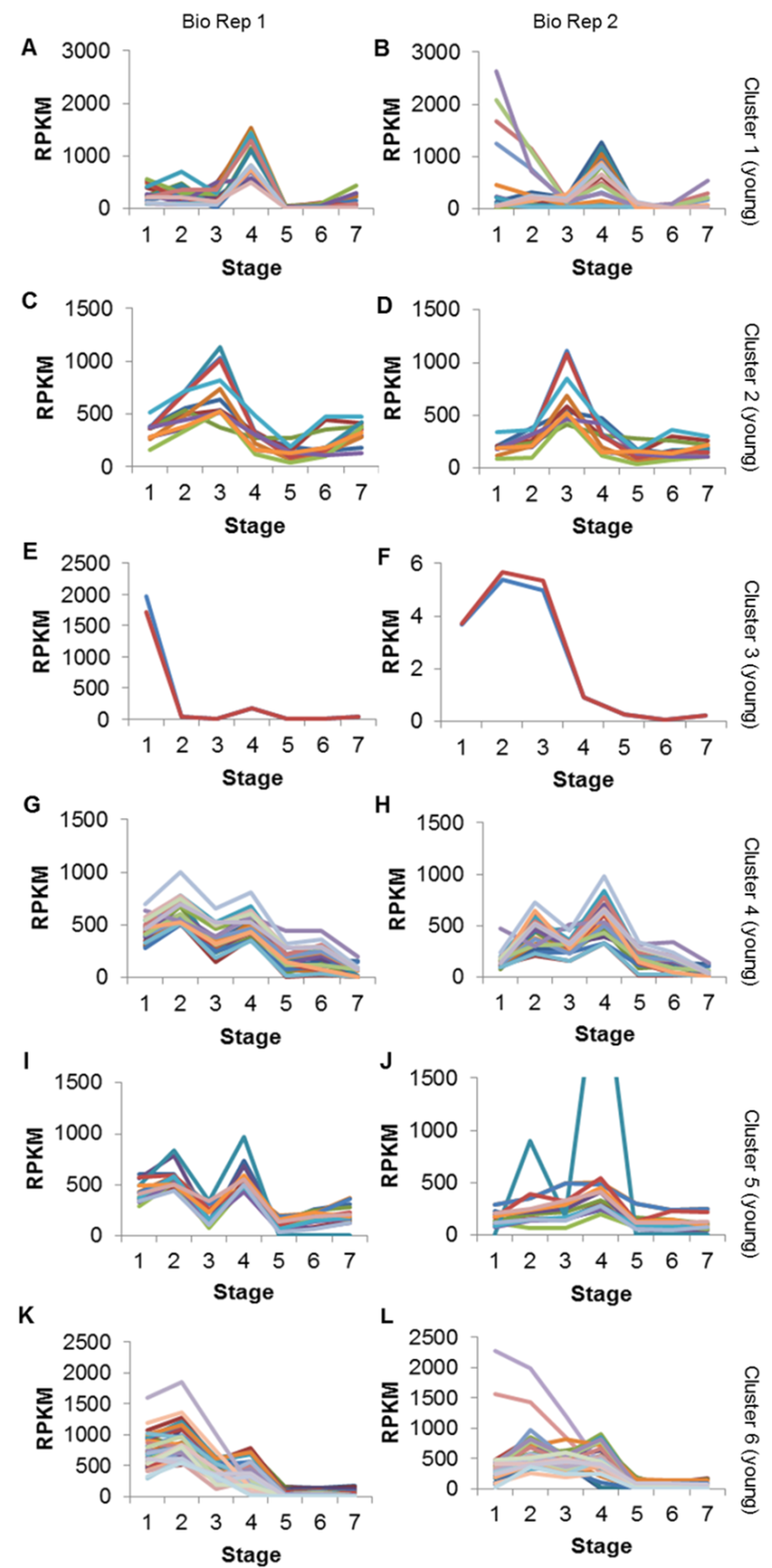

Supplement: Figure S1 — Clusters of gene models with high expression in young seed stages. Clusters produced using Biological Replicate 1 data (left column) depict gene models with RPKM≥500 in at least one of four young seed stages, and RPKM <500 in all three older stages. The right column shows the same gene models with Biological Replicate 2 data. Cluster numbers (determined by MeV) are shown at the far right. A and B are repeated from Figure 1C and 1D; K and L are repeated from Figure 1A and 1B. Note the different scales used by E and F. The Y axis of J has been set at 1500, cutting off the blue line, which reaches an RPKM of over 2700 at 5–6 mg whole seed. Stages are numbered in order on the x axis: 4 DAF whole seed, 12–14 DAF whole seed, 22–24 DAF whole seed, 5–6 mg whole seed, 100–200 mg cotyledon, 400–500 mg cotyledon, dry whole seed. (TIF) [file pone.0059270.s001.tif]

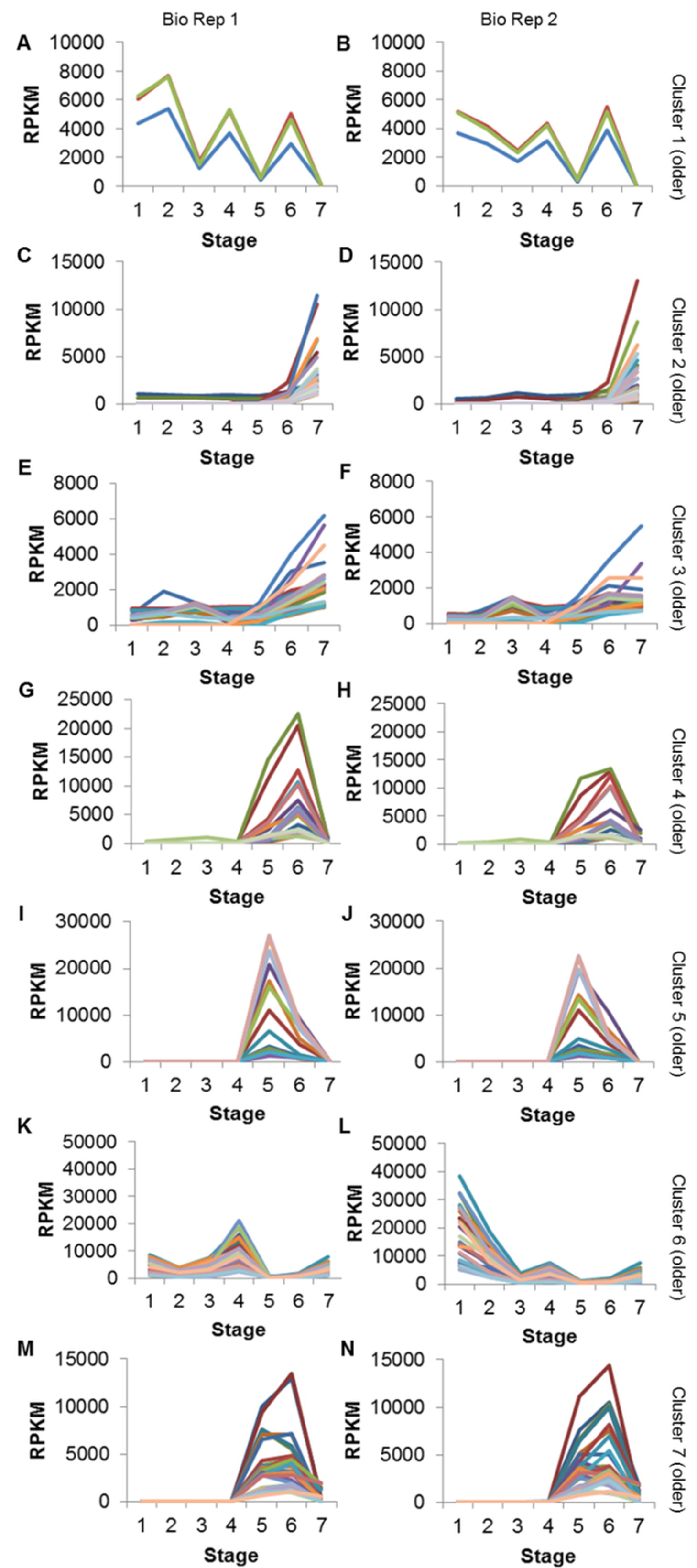

Supplement: Figure S2 — Clusters of gene models with high expression in older seed stages. Clusters produced using Biological Replicate 1 data (left column) depict gene models with RPKM≥1000 in at least one of three older seed stages. The right column shows the same gene models with Biological Replicate 2 data. Cluster numbers (determined by MeV) are shown at the far right. C and D are repeated from Figure 1G and 1H; M and N are repeated from Figure 1E and 1F. Stages are numbered in order on the x axis: 4 DAF whole seed, 12–14 DAF whole seed, 22–24 DAF whole seed, 5–6 mg whole seed, 100–200 mg cotyledon, 400–500 mg cotyledon, dry whole seed. (TIF) [file pone.0059270.s002.tif]

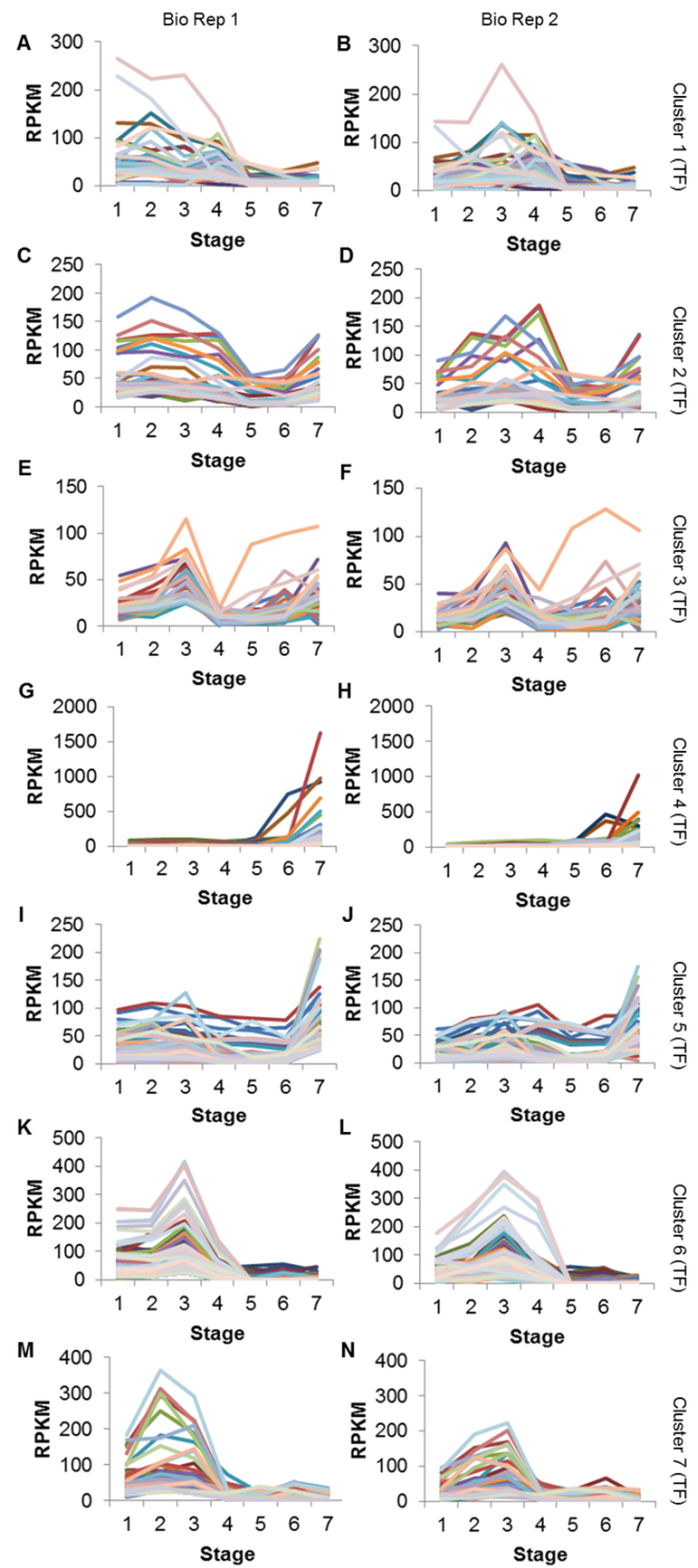

Supplement: Figure S3 — All clusters of transcription factor gene models. Clusters produced using Biological Replicate 1 data (left column) depict gene models annotated as transcription factors with RPKM≥25 in at least one of seven stages of seed development. The right column shows the same gene models with Biological Replicate 2 data. Cluster numbers (determined by MeV) are shown at the far right. Annotations were derived from PFAM and/or the NCBI non-redundant database. Stages are numbered in order on the x axis: 4 DAF whole seed, 12–14 DAF whole seed, 22–24 DAF whole seed, 5–6 mg whole seed, 100–200 mg cotyledon, 400–500 mg cotyledon, dry whole seed. (TIF) [file pone.0059270.s003.tif]
